# What Intervention Elements Drive Weight Loss in Blended-Care Behavior Change Interventions? A Real-World Data Analysis with 25,706 Patients

**DOI:** 10.3390/nu14142999

**Published:** 2022-07-21

**Authors:** Felix Schirmann, Philipp Kanehl, Lucy Jones

**Affiliations:** 1 Oviva AG, 10117 Berlin, Germany; philipp.kanehl@oviva.com; 2 Oviva AG, London SE1 9RS, UK; lucy.jones@oviva.com

**Keywords:** blended-care behavior change interventions, weight loss, coaching, self-monitoring, self-management, education

## Abstract

Background: Blended-care behavior change interventions (BBCI) are a combination of digital care and coaching by health care professionals (HCP), which are proven effective for weight loss. However, it remains unclear what specific elements of BBCI drive weight loss. Objectives: This study aims to identify the distinct impact of HCP-elements (coaching) and digital elements (self-monitoring, self-management, and education) for weight loss in BBCI. Methods: Long-term data from 25,706 patients treated at a digital behavior change provider were analyzed retrospectively using a ridge regression model to predict weight loss at 3, 6, and 12 months. Results: Overall relative weight loss was −1.63 kg at 1 month, −3.61 kg at 3 months, −5.28 kg at 6 months, and −6.55 kg at 12 months. The four factors of BBCI analyzed here (coaching, self-monitoring, self-management, and education) predict weight loss with varying accuracy and degree. Coaching, self-monitoring, and self-management are positively correlated with weight losses at 3 and 6 months. Learn time (i.e., self-guided education) is clearly associated with a higher degree of weight loss. Number of appointments outside of app coaching with a dietitian (coach) was negatively associated with weight loss. Conclusions: The results testify to the efficacy of BBCI for weight loss-with particular positive associations per time point-and add to a growing body of research that characterizes the distinct impact of intervention elements in real-world settings, aiming to inform the design of future interventions for weight management.

## 1. Introduction

### 1.1. Background

Blended-care behavior change interventions (BBCI) are a combination of digital care and coaching by health care professionals (HCP) that have proven themselves effective for weight management [1,2,3,4,5,6,7,8]. BBCI comprise the remote delivery of care with digital communication tools (e.g., an app-based chat), actual care by HCP (e.g., app-feedback by a dietitian), as well as self-administered digital intervention elements (e.g., self-monitoring through photo-logging of meals). Accordingly, BBCI are distinct from stand-alone digital care, where patients are not supported by an HCP (e.g., chat bots) [9]. BBCI have enormous potential to tackle the rising obesity prevalence rates through increasing the accessibility of care, whilst being scalable and economical, and relieving the pressure on scarce HCP-resources [10,11]. In particular, their potential for large-scale weight management interventions is promising and unprecedented [12]. Additionally, qualitative research highlights that patients appreciate the easy integration of BBCI into their daily routines and the high levels of flexibility [3,13], driving overall adherence [1]. Constantly evolving technology offers a host of digital care approaches for nutrition-related diseases that can be combined with HCP-care in multiple ways, ranging from remote monitoring of patients’ parameters and peer-group support chats to telehealth sessions with doctors [11]. As a consequence, BBCI are diverse and complex, complicating research on their effectiveness. Against this backdrop, systematic reviews and meta-analyses on the effectiveness of BBCI for weight management report mixed results—mostly attributed to the heterogeneity of studies—and unanimously call for more rigorous research [1,5,6,7,8]. Metzendorf et al. [14] stated that the theoretical underpinnings and care components needed clarification. In a similar vein, Chatterjee et al. [15] called for analysis of frameworks, procedures, and methods, whilst Duan et al. [7] advocated for improving intervention designs.

One way to improve the understanding of BBCI is to analyze their components, i.e., their distinct intervention elements along with their impact for weight loss. Identifying and quantifying the potent intervention elements requires the segmentation, differentiation, and standardization of the elements of interventions. The behavior change taxonomy is a conceptual framework offering this; characterizing 93 distinct behavior change techniques used in complex interventions [16]. Various systematic reviews apply the behavior change taxonomy to identify and gauge the effects of intervention elements in BBCI [6,17,18,19]. Due to the widespread usage of the behavior change taxonomy, this approach is conducive as it aids standardization. The taxonomy does not however account for the origin of the intervention element, i.e., HCP versus digital, thereby neglecting a potentially impactful distinction. Thus, existing evidence is categorized and reviewed here in accordance with the distinct BBCI in the focus of this study—that is, HCP-delivered elements (coaching) and digital elements (self-monitoring, self-management, and education) for weight loss.

The HCP themselves, the feedback they provide, as well as the rapport they establish with their patients have all been identified as critical to the success of interventions [20,21]. Operationalizing the HCP element for research purposes is challenging due to the complexity of the human interaction. One option is to examine HCP’s use of intervention methods, such as behavior change techniques and motivational interviewing. However, such studies yielded mixed and partially inconclusive results, likely linked to this complexity [22]. For example, feedback on behavior by the HCP is a very frequent intervention element, yet varying definitions, intensities, and delivery styles complicate comparisons [21]. For simplification and quantification purposes, the interaction frequency between HCP and their patients (e.g., number of sessions or number of chat messages) can be used. Following this approach, Painter et al. [23] quantified the small, but positive, contribution of individual HCP-patient messages on patients’ adherence (food logging, meal logging, physical activity). The mode of communication between the HCP and their patients (e.g., face to face, app-chat or telephone) seems to have no impact on outcomes, with face-to-face and remote interventions yielding comparable results [24]. However, patient characteristics might influence the perceived HCP elements, e.g., in the formation of rapport, influencing the efficacy of BBCI. In fact, research has identified patient-specific barriers, such as technology illiteracy [25], age [26], and socioeconomic status [27], although the distinct impact of these factors remains unclear. In addition, patients’ perceptions of the intervention and its progress could impact BBCI outcomes. For example, Troinieri et al. [28] showed that early weight loss was the strongest predictor of final weight loss. Early perceived success or failure might influence a patients’ motivation and thereby determine later outcomes.

With regard to digital elements, general usage frequency of the intervention device (e.g., an app) has been shown to predict higher weight loss [12,29], testifying to the high importance of adherence for effective weight management. Self-monitoring frequency is often used as an indicator of adherence with a host of studies demonstrating that this intervention element drives weight loss [6,30,31,32,33]. Specifically, Raber et al. [31] point out that high as well as low intensity levels of self-monitoring are effective. Multiple digital tools (websites, mobile applications, wearables, electronic scales; see [33]) are available for self-monitoring, with mobile applications enabling photo-based meal logs. Meal logging has been shown to be associated with higher weight loss in two retrospective analyses [23,34].

One reason for focusing on digital intervention elements is that they enable self-care through assisting with self-management in patients’ everyday lives. A recent study by Dwibedi et al. [35] demonstrated that digital self-management, even without HCP-involvement, was beneficial for body weight, blood glucose levels, systolic blood pressure, and insulin resistance in patients with type 2 diabetes. Assisted self-management with digital means can be realized in multiple ways. One frequent feature of digital tools is task/goal setting, allowing patients, for example, to set and monitor goals for daily physical activity [6]. Importantly, tasks/goals can be set for behaviors and (clinical) outcomes, depending on the feature and or the intervention design.

Education elements mainly aim to foster health-literacy for nutrition-related diseases and their consequences. The rationale is that altered or additional knowledge will drive the cognitive and emotional reappraisal of patients’ behaviors, leading to a decrease in unhealthy behaviors, an increase in health behaviors, and ultimately in the formation of new habits. While the efficacy of education aiming at self-reflection has been criticized due to its over-reliance on cognitive processes and their questionable link to behaviors [36], education is frequently used in BBCI, featuring prominently in 30% of app-based interventions [21]. Painter et al. [23] quantified the effect of education, operationalized as the completion of digital lessons, to be a loss of 0.14 kg per completed lesson.

While coaching, self-monitoring, self-management, and education seem to contribute to weight loss, their individual effects need scientific scrutiny. Because intervention elements are often administered in combination or clusters [6], the analysis of their individual contribution to weight loss is methodologically challenging. In addition, the effects of intervention elements (and their associated technological features) could potentially only unfold when combined. On this point, Antoun et al. [21] illustrated that individual features or their total number were not associated with weight loss. Due to the complexity of BBCI in real-world care settings, several (digital and human) factors will create intercorrelated, higher-order factors, whose impact—in isolation and in combination—has yet to be analyzed in detail.

### 1.2. Goal of This Study

Against this backdrop, the aim of this study was to determine the individual effect of intervention elements in BBCI on weight losses at 3, 6, and 12 months in a real-world setting. For this, long-term data (>12 months) from 25,706 patients treated at a digital behavior change provider for nutrition-related conditions were analyzed retrospectively. HCP- elements (coaching) and digital elements (self-monitoring, self-management, education) were analyzed separately. We hypothesized that higher amounts of all four intervention elements would drive higher weight loss.

## 2. Materials and Methods

### 2.1. Study Design

A retrospective linear regression (Bayesian ridge regression with standardized targets and covariates) was performed. One HCP- (coaching) and three digital intervention elements (self-monitoring, self-management, and education) at months 1–3 were used as predictors for 3-, 6-, and 12-month weight loss.

### 2.2. Participants

Data from 25,706 patients (17,749 female, 7880 male, 77 unspecified; mean age: 47.3 years, SD 10.96), who received BBCI at a digital behavior change provider (Oviva AG) for prevention or therapy of nutrition-related conditions in the UK, Germany, and Switzerland, were analyzed after patients had completed an intervention period of up to 12 months. Patient data was included in the analysis if they used the Oviva app and had weight data available at baseline, in addition to at least one outcome weight at 1, 3, 6 or 12 months. All patients acknowledged and confirmed Oviva’s terms and conditions and privacy policies (per country) and thus consented to their anonymized data being used for research.

### 2.3. Application and Delivery of BBCI

BBCI, featuring HCP- and digital elements, were made accessible for participants, for at least 12 months. The HCP- intervention element (coaching) was delivered in line with the respective national guidelines for the particular nutrition-related disease by certified health coaches and/or dietitians. The number of sessions, timing of sessions, and session lengths varied per country and per care pathway, respectively. For interaction with the coach and delivery of the digital BBCI (self-monitoring, self-management, and education), the Oviva app was used (available for Android phones and Apple phones). With the app, patients self-monitored by using photos to log meals and by logging other health-related behaviors (such as physical activity). The app allowed for self-management with a task/goal setting feature (including reminders and an overview of completed tasks). Lastly, condition-specific education materials (texts, videos, podcasts) were delivered via the app.

### 2.4. Data Collection

Data on all four BBCI were continuously and automatically collected in the secure system of the digital behavior change provider. The three digital BBCI (self-monitoring, self-management, and education) were registered via the app of the provider whenever patients applied them. For example, the event of a patient taking a photo of their meal via the app created a specific log entry, allowing for continuous data collection on meal-logging (self-monitoring). The HCP- intervention element (coaching) was registered via the provider’s patient management system that logged all appointments and coach messages.

### 2.5. Measures and Statistical Analysis

Clinical weight outcomes were predicted at t∈ [1, 3, 6, 12] months after an initial coaching session with a dietitian had taken place. More specifically, the relative weight change was predicted, $\delta_{t,i}=w_{t,i}{}_{}$/$w_{0,i}-1$, where $w_{t,i}$ is the body weight at time t  of user i.

For the independent variables, seven covariates were used, based on the four intervention elements specified above. With the exception of self-management, intervention elements were operationally defined by two metrics each, to account for the variability of definitions of BBCI found in the literature and to make the measurements more robust (see column ‘covariate/aspect of intervention element’ in Table 1).

The model is a multiple regression, where the relative weight change $\delta_{t,i}$ is predicted from the vector of normalized covariates $\underline{c_{\tau ,i}}$, where τ≤t.

The model is:

$$\delta_{t,i}={\underline{\alpha }}_{t}{}^{}\cdot \underline{c_{\tau ,i}}+\beta_{t}+\epsilon_{t,i}$$

where the residuals $\epsilon_{t,i}$ are assumed normally distributed with zero mean and unknown standard deviation $\sigma_{t}$, i.e.,

$$\epsilon_{t,i}\;∼N\left(0,\;\sigma_{t}\right)\;$$

The parameters ${\underline{\alpha }}_{t}{}^{}$, $\beta_{t}$ and $\sigma_{t}$ are estimated for each outcome period t using Bayesian inference utilizing the open-source Python library PyMC3.

Finally, the parameters were regularized by the following weekly informative prior distributions:

$${\underline{\alpha }}_{t}{}^{}∼\underline{N}\left(0,\;0.05\right),\;\beta_{t}∼N\left(0,\;0.05\right),\;\mathrm{and}\;\sigma_{t}∼HalfNormal\left(0.05\right).$$

Since any engagement is volunteered by the users, the inferred parameters do not have a causal interpretation. Moreover, behaviors associated with digital intervention elements (e.g., meal logging) vary greatly across individual users, which leads to differing cohorts for each considered time period t. In particular, 6- and 12-months data for parts of the sample considered was not available due to missing data.

## 3. Results

### 3.1. Overall Weight Loss

Table 2 shows average relative and absolute weight loss data for the cohort selected here at the different time points.

**Figure 1:**
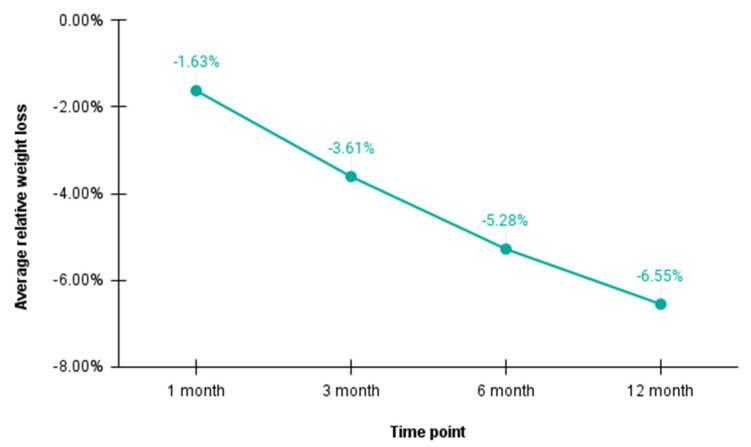
Average relative weight losses (in percent. compared to average baseline weight) at 1, 3, 6, and 12 months.

Figure 1 depicts patients’ average relative weight loss over the course of 12 months.

### 3.2. Contribution of Intervention Elements to Weight Loss

**Figure 2:**
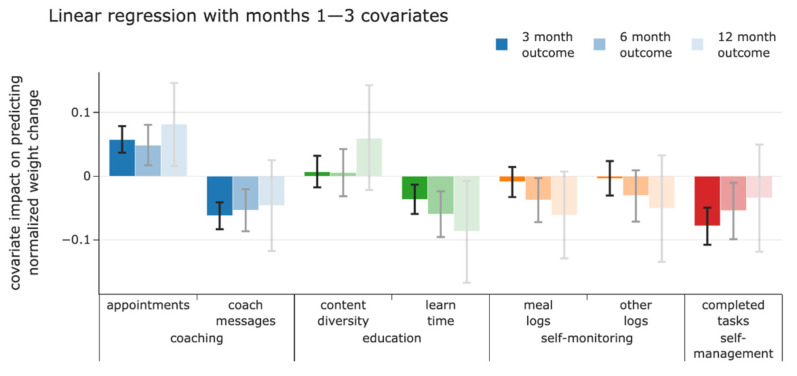
Contribution of the HCP-element (blue) and digital intervention elements (green, yellow, red) to weight losses at 3, 6, and 12 months (including 95% credibility intervals for estimated coefficients based on their posterior probabilities). Ordinate units are in standard deviation of the relative weight change divided by the standard deviation of each covariate, respectively. For example, a user with 1 standard deviation above average learn time during the first 3 months, is predicted to achieve 0.04, 0.06, or 0.09 standard deviations above average weight loss at months 3, 6, or 12, respectively.

The four intervention elements and their subordinated aspects predicted weight losses at 3, 6, and 12 months to varying degrees (see Figure 2).

#### 3.2.1. HCP Element: Coaching

Number of appointments, the first aspect of the intervention element coaching, was negatively associated with weight loss at 3 months (95% credibility interval of posterior mass CI [0.04, 0.08]), 6 months (95% CI [0.02, 0.08]), and 12 months (95% CI [0.02, 0.15]). Number of coach messages sent, the second aspect of the intervention element coaching, was positively associated with weight loss at 3 months (95% CI [−0.08, −0.04]), 6 months (95% CI [−0.09, −0.02]), and 12 months (95% CI [−0.12, 0.02]).

#### 3.2.2. Digital Element: Self-Monitoring

Number of meal logs, the first aspect of the intervention element self-monitoring, was positively associated with weight loss at 6 months (95% CI [−0.07, 0.0]) and 12 months (95% CI [−0.13, 0.01]). At 3 months (95% CI [−0.03, 0.02]), we only yield 65% probability that the coefficient is negative with expectation (−0.01) (see Figure 2). Similarly, for the number of other logs, the second aspect of the intervention element self-monitoring, a positive association with weight loss was observable, but with an overall lower expectation (3 months (95% CI [−0.03, 0.02]), 6 months (95% CI [−0.07, 0.01]), and 12 months (95% CI [−0.14, 0.03]).

#### 3.2.3. Digital Element: Self-Management

Number of completed tasks was positively associated with weight loss at 3 months (95% CI [−0.11, −0.05]) and 6 months (95% CI [−0.10, −0.01]). At 12 months, we obtain a 72% probability that the correlation with weight loss is positive with expectation 0.03 (95% CI [−0.13, 0.06]) (see Figure 2).

#### 3.2.4. Digital Element: Education

Based on visual inspection (see Figure 2), the association of content diversity, the first aspect of the intervention element education, with weight loss was likely small with inconclusive direction (3 months (95% CI [−0.02, 0.03]), 6 months (95% CI [−0.03, 0.04]), and 12 months (95% CI [−0.02, 0.14]). Learn time, the second aspect of the intervention element education, was positively associated with weight loss at 3 months (95% CI [−0.06, −0.01]), 6 months (95% CI [−0.09, −0.02]), and 12 months (95% CI [−0.17, −0.0]).

## 4. Conclusions

### 4.1. Principal Results and Comparison with Prior Work

The obtained results indicate a complex relationship between the four intervention elements (coaching, self-monitoring, self-management, education) and weight losses at 3, 6, and 12 months.

First, intervention elements have different effects per time point. For example, self-monitoring within the first three months (i.e., both aspects: meal logs and other logs) correlates stronger with more distant weight-loss outcomes, indicating positive returns of early self-monitoring at 6 and 12 months. Time-dependency is frequently evidenced in BBCI in the form of a decline in patients’ adherence in the course of the treatment and, as a consequence, a reduction of the frequency of self-administered intervention elements [21]. Accordingly, the general usage frequency of digital devices used in BBCI has been shown to predict higher weight loss [12,28]. Here, however, time-dependency of different intervention elements is demonstrated for weight loss per treatment phase. Self-management is clearly positively associated with weight loss but decorrelates for more distant outcomes. This is symptomatic as task completion acts mainly as a mediator for the other intervention elements.

Second, certain intervention elements have a positive correlation with weight loss at all time points, testifying to their time-independence. The positive correlation of learn time at 3, 6, and 12 months indicates that early-stage dietary education facilitates positive weight outcomes throughout long-term care. For learn time, the positive correlation even increases over time, indicating that early-stage education could act as a foundational driver of later weight loss.

Third, differing operational definitions of intervention elements affect research results. In this study, education was quantified with two metrics (content diversity and education) and yielded differing results per metric. Learn time was positively associated with weight loss throughout the considered timespan, whereas the association of content diversity was negligible or negatively associated. The heterogeneity of operational definitions and the resulting incomparableness (and possible irreproducibility) of findings is frequently pointed out as an impediment in BBCI research [1,5,6,7,8]. Detailed documentation as well as standardization of operational definitions for intervention elements (e.g., regarding user-friendly design for digital intervention elements) to enable comparability between studies will be conducive for future research.

Fourth, the HCP element coaching produced contradictory effects, which runs counter to previous research [20,21]. Number of coach messages was positively associated with weight loss, but the number of appointments was negatively associated. One possible explanation for this lies in the utilization of coaching based on case severity. In general, more complex cases will receive longer treatment periods and a higher number of appointments. The resulting positive correlation of case complexity and number of appointments in this sample could explain the counter-intuitive results obtained here. In addition, these patients could rely on appointments without utilizing the self-administered intervention elements, thereby not benefitting from the holistic effect of BBCI. Further subgroup analyses to establish the specific association of certain BBCI with particular groups of patients are needed for corroboration.

Fifth, there are seemingly unassociated aspects of intervention elements—e.g., content diversity at 3 and 6 months. Their lack of predictive value for weight loss might be attributed to the intercorrelation of predictors in the model used, resulting in their relative insignificance when analyzed independently of the corresponding other aspect of the respective intervention element (i.e., learn time for content diversity as part of coaching). While it would be premature to discard these aspects, further analyses with refined operational definitions (see above), alternative predictive modeling approaches, and different samples might aid in understanding their individual contribution.

### 4.2. Limitations

The sample used in this study was highly diverse, e.g., patients had different nutrition-related diagnoses and participated in a variety of treatment programs with different durations, coaching intensities, and treatment goals. In addition to this, the aim of this study was to determine the individual effect of intervention elements in BBCI on weight loss; however, this fine-grained mode of analysis might be hampered by intervention elements acting synergistically. Their holistic effect on weight loss could be more than the sum of their parts. Furthermore, predictors in the linear regression model used here were highly intercorrelated, reducing the interpretability of the impact of the individual intervention elements. Lastly, it is well established that patients with low weight loss are more likely to churn or drop out from treatment over time [37]. Accordingly, these churned patients will not have contributed data at later time points (e.g., 12 months). The regression model does not account for this idiosyncrasy of the sample.

### 4.3. Conclusion and Future Research

The results are instructive as they inform an emerging body of research that strives to single out the individual contribution of intervention elements for weight loss—with the aim to inform BBCI monitoring and design. Future research could apply the linear regression model along with the operational definitions of intervention elements used here to different data sets to probe the consistency of the findings and to contribute to the standardization of methodological approaches in the field. In addition, refining the model by using alternative predictors, such as continuous data from wearables [38], could advance the understanding of weight loss in real-world settings. In general, large-scale analyses of real-world data sets that capitalize on the richness of digital devices as data sources will likely aid the understanding of the uptake, adherence, patient-friendliness, and clinical outcomes of BBCI [39]. For example, new modes of analysis are emerging: millions of app-derived meal logs can be used to analyze a patient’s meal composition, potentially enabling automated diet recommendations [40]. Tailoring [41] and personalization [42,43] of BBCI in particular will profit from subgroup analyses of large data sets. In addition, research is needed on how BBCI can improve existing treatment approaches and nutrition concepts for weight management [44]. Future studies also need to determine which intervention elements are best delivered by HCP and which can be delegated to digital devices to make the best use of scarce HCP resources. Standardization of intervention elements and associated operational definitions will be key to enable comparability and generalizability of studies overall. Finally, clinical trials, especially randomized controlled trials, will need to be conducted to drive BBCI research and corroborate findings from real-world data analyses such as the study at hand. The resulting evidence base will be crucial to inform decision-making within the growing number of healthcare systems that embrace BBCI [45]. To conclude, this study adds to a growing body of research on the efficacy of BBCI for weight loss and documents their multifaceted impact per treatment phase in a real-world care setting.

## Figures and Tables

**Table 1:** Description of independent variables used in the multiple regression.

Intervention Element	Covariate/Aspect of Intervention Element	Description	Unit
Coaching	Appointments	Number of live sessions with a coach, either via phone or face to face, within the period (0, *τ*).	1
	Coach messages	Number of messages sent to the patient by the coach via the Oviva app within the period (0, *τ*).	1
Self-Monitoring	Meal logs	Number of meals tracked via text and/or photo within the Oviva app and the period (0, *τ*).	1
	Other logs	Number of activities, symptoms or measurements (e.g., weight, blood glucose) tracked within the Oviva app and the period (0, *τ*).	1
Self- Management	Completed tasks	Number of completed tasks assigned by the coach or the patient, e.g., track your meal today, make 5000 steps, etc., within the period (0, *τ*).	1
Education	Content diversity	Number of unique learn units that have been opened at least once within the period (0, *τ*).	1
	Learn time	Total time media content was open in the Oviva app and within the period (0, *τ*).	minutes

**Table 2:** Average relative and absolute weight loss (in percent and kg, compared to average baseline weight) at 1, 3, 6, and 12 months.

Time Point	Average Baseline Weight (0 Month) ± Standard Deviation	Average Relative Weight Loss ± Standard Deviation	Average Weight Loss ± Standard Deviation	Number of Patients (*n*)
1 month	106.7 ± 21.4 kg	−1.63 ± 5.94%	−1.89 ± 7.82 kg	15,012
3 months	106.6 ± 21.3 kg	−3.61 ± 5.82%	−4.02 ± 7.82 kg	9526
6 months	106.5 ± 21.1 kg	−5.28 ± 6.94%	−5.82 ± 9.10 kg	4204
12 months	106.5 ± 19.7 kg	−6.55 ± 8.22%	−7.22 ± 9.67 kg	979

## Data Availability

The data are not publicly available due to privacy restrictions.

## Abbreviations

BBCI	blended-care behavior change interventions
HCP	health care professionals
CI	credibility interval
